# The Role of Hexokinases in Epigenetic Regulation: Altered Hexokinase Expression and Chromatin Stability in Yeast

**DOI:** 10.21203/rs.3.rs-3899124/v1

**Published:** 2024-01-30

**Authors:** Srinivasu Karri, Quinn Dickinson, Jing Jia, Haiyun Gan, Zhiquan Wang, Yibin Deng, Chuanhe Yu

## Abstract

**Background** . Human hexokinase 2 ( *HK2* ) plays an important role in regulating Warburg effect, which metabolizes glucose to lactate acid even in the presence of ample oxygen and provides intermediate metabolites to support cancer cell proliferation and tumor growth. *HK2* overexpression has been observed in various types of cancers and targeting *HK2* -driven Warburg effect has been suggested as a potential cancer therapeutic strategy. Given that epigenetic enzymes utilize metabolic intermediates as substrates or co-factors to carry out post-translational modification of DNA and histones in cells, we hypothesized that altering *HK2* expression-mediated cellular glycolysis rates could impact the epigenome and, consequently, genome stability in yeast. To test this hypothesis, we established genetic models with different yeast hexokinase 2 ( *HXK2)* expression in *Saccharomyces cerevisiae* yeast cells and investigated the effect of *HXK2* -dependent metabolism on parental nucleosome transfer, a key DNA replication–coupled epigenetic inheritance process, and chromatin stability. **Results** . By comparing the growth of mutant yeast cells carrying single deletion of *hxk1Δ* , *hxk2Δ* , or double-loss of *hxk1Δ hxk2Δ* to wild-type cells, we demonstrated that *HXK2* is the dominant *HXK* in yeast cell growth. Surprisingly, manipulating *HXK2* expression in yeast, whether through overexpression or deletion, had only a marginal impact on parental nucleosome assembly, but a noticeable trend with decrease chromatin instability. However, targeting yeast cells with 2-deoxy-D-glucose (2-DG), a *HK2* inhibitor that has been proposed as an anti-cancer treatment, significantly increased chromatin instability. **Conclusion** . Our findings suggest that in yeast cells lacking *HXK2* , alternative *HXK* s such as *HXK1* or glucokinase 1 ( *GLK1* ) play a role in supporting glycolysis at a level that adequately maintain epigenomic stability. While our study demonstrated an increase in epigenetic instability with 2-DG treatment, the observed effect seemed to occur independently of Hxk2-mediated glycolysis inhibition. Thus, additional research is needed to identify the molecular mechanism through which 2-DG influences chromatin stability.

## Background

Hexokinases (Hks), the rate-limiting enzyme responsible for glucose metabolism, catalyzes the phosphorylation of glucose to glucose-6-phosphate (Glc-6-P). Glc-6-P subsequently enters glycolysis or alternative metabolic pathways, serving as a vital substrate for adenosine triphosphate (ATP) production and the biosynthesis of various metabolites. There are four Hk isozymes (Hk1-4) in humans. And each has a different tissue and organ distribution, as well as distinct metabolic functions (1). Expression of some *Hks* is associated with worse prognosis in several tumor types. For example, *HK1* plays an oncogenic role in bladder cancer (2), and *HK2* enhances live cancer stemness (3). High *HK2* expression has been linked to a variety kind of cancer and is associated with poor overall survival in cancer patients (4–6). For these reasons, targeting Hks has been suggested as a potential strategy for cancer therapy (7, 8). In support of this approach, deleting *HK2* can decrease cancer cell proliferation without prominent side effects in animal models, suggesting *HK2* is a promising target for cancer therapy (5). However, the mechanism by which altered *HK* expression on other non-glycolysis cellular functions is poorly understood. To learn more about the utility of targeting *HK*s, it is important to characterize the effects of modulating *HK* expression on eukaryotic cells.

As a key node in glucose metabolism and the main producer of energy, acetyl-coenzyme A (Acetyl-CoA) is also the substrate of globe histone acetylation (9). Furthermore, Epigenetic enzymes employ several glucose metabolic intermediates as co-factors to carry out post-translational modifications of DNA and histones (10). Thus, altering the expression of cellular metabolism genes, such as the *HKs*, may change global epigenetic states. This, in turn, may create conditions that promote cancer cell proliferation, consistent with *HKs* overexpression effects in cancer cells.

In this study, we investigated how abnormal yeast Hexokinase 2 (*HXK2)* expression affects epigenetics and genomic instability in a simple, genetically tractable model system for eukaryotic cell function: the budding yeast *Saccharomyces cerevisiae*. In this organism, three genes encode proteins that phosphorylate glucose to Glc-6-P: *HXK1, HXK2*, and the glucokinase gene *GLK1*. *HXK2* appears to play the main role in glucose phosphorylation in vivo (11, 12). Therefore, we investigated the effects of *HXK2* deletion and overexpression on parental histone H3-H4 transfer, an essential step in epigenetic inheritance (13). We hypothesized that the DNA-replication coupled epigenetic inheritance process is altered because DNA replication is an energy consuming process. We found both types of altered *HXK2* expression had only a minor effect on parental histone H3-H4 distribution on replicating chromatin and appeared to decrease chromatin instability. However, 2-deoxy-D-glucose (2-DG), an Hxk2 inhibitor that has been used as a cancer therapy (14), increased chromatin instability in yeast. The data indicates that genetically altered Hxk2 expression has only a minor impact on the epigenome and genomic stability. However, the pharmacological use of the Hxk2 inhibitor 2-DG treatment does lead to an increase in chromatin instability.

### Materials and methods

## Yeast strains, plasmids, and growth conditions

*S. cerevisiae* yeast strains were grown in YPD (2% peptone, 1% yeast extract, 2% glucose) or yeast nitrogen base minus uracil (AA-Ura) media, to prevent eviction of plasmids at 30°C (15). Yeast strains used in this study are listed in Supplemental Table 1. Plasmids were transformed into yeast strains using the standard lithium acetate transformation method (16). AA-Ura or YPD plates containing 2% glucose were supplemented with 20% 2-DG (20 g 2-DG dissolved in 100 ml sterile water) to a final concentration of 0.2% w/v.

#### HXK cloning and yeast strain construction

To amplify *yHXK1* and *yHXK2* from yeast genomic DNA, we employed specific primer pairs (Kpn1-*HXK1*/Xba1-*HXK1* and Kpn1-*HXK2*/Xba1-*HXK2*; primer sequences provided in Supplemental Table 2). Following amplification, the resulting DNA fragments were gel-purified using a Qiagen gel purification kit and subsequently cleaved with Kpn1 and Xba1 restriction enzymes. The resulting fragments were inserted into the yeast expression vector PSF-TEF1-URA3 (OGS534, Milipore-Sigma), exploiting the corresponding restriction enzyme sites.

The yeast-codon optimized human *HK* genes *hHK1* and *hHK2* were synthesized directly, with the sequences described in the Supplemental Data. These gene fragments were enzymatically cut using the restriction enzymes EcoRV and XhoI and integrated into the PSF-TEF1-URA3 vector, utilizing the matching restriction enzyme sites.

To introduce the *HXK*-containing vectors and vector controls into yeast, we followed the standard lithium acetate transformation protocol. Colonies were selected on yeast synthetic complete supplement mixture minus uracil (SC-Ura) plates.

## Immunoblotting assay

To monitor the levels of histone marks in yeast strains, we performed whole cell extraction using tris-buffered saline buffer (20 mM Tris pH 7.5, 150 mM NaCl) with proteasomal inhibitors. Briefly, mid-log-phase cells (5 OD at 600 nm) were harvested by spinning cultures at 5000 rpm then washing the pellets with water, heating them at 95° C for 3 min, and suspending them in 50 ul tris-buffered saline buffer with 2 mM phenylmethylsulfonyl fluoride. Cells were physically broken by glass bead–beating them for 2 cycles (30 sec per cycle). Proteins were then solubilized in 50 ul 2x SDS sample buffer [Tris-Cl (pH 6.8), 4% (w/v) sodium dodecyl sulfate (SDS; electrophoresis grade), 0.2% (w/v) bromophenol blue, 20% (v/v) glycerol]. Insoluble debris was separated from supernatant and removed by spinning the samples at the maximum speed. Soluble proteins (the supernatant) were separated by SDS-PAGE and probed with different antibodies [anti-γ-H2A antibody (ab15083 Abcam), H3K4me3 (ab8550 Abcam), H3K36me3 (ab9050 Abcam), H3K56Ac (mc1681(17)), Sir2 (18), H3K27Ac (07-360 Millipore), and H3K5,8,12Ac (C15410021 Diogenode)].

## Cell cycle analysis

Yeast strains were grown in YPD medium at 30° C until the cells reached mid-log phase (~ 0.6 OD at 600 nm), at which time they were treated with 2-DG (0.2% w/v). A total of ~ $1X10^7$ cells were harvested at various time points by centrifugation, washed with water, and fixed with 95% ethanol overnight at 4° C. Fixed cells were collected by centrifugation, washed with 50 mM sodium citrate buffer (pH 7.5), and resuspended in 500 µL sodium citrate buffer (pH 7.5) with RNAase A (0.025 mg/mL). After cells were incubated for 1 h at 50° C, 25 µL proteinase K (20 mg/mL) was added to the cell suspension and the mixture was incubated at 50° C for another hour. Cells were subsequently stained overnight at 4° C using propidium iodide at a final concentration of 0.02 mg/mL. Finally, samples were analyzed in a fluorescence-activated cell sorting (FACS) flow cytometer (BD Fortessa cytometer). A minimum of 50,000 cells per sample were acquired.

**Analysis of silencing-loss at the HML locus using the CRASH assay**

The yeast strains WT (cyc1250), *hxk2Δ* (cyc1253), WT + *PSF-yHXK2* (cyc1247), and *hxk2Δ + PSF-yHXK2* (cyc1251) were used to measure the apparent silencing-loss rate at the *HML* locus. Briefly, 10 colonies of each strain were grown separately overnight in SC media, diluted to 0.01 OD at 600 nm in SC media, and grown for 5 hours at 30° C. Cells were treated with 20 μM nicotinamide for the green fluorescent protein (GFP) + control; cells were grown in hygromycin (200 μg/ml) for the red fluorescent protein (RFP) + control. The apparent silencing-loss rate at the *HML* locus was calculated by dividing the number of RFP + GFP + cells (cells that have recently undergone Cre-mediated recombination express GFP but not RFP) by the total number of cells with the potential to lose silencing (RFP + GFP- and RFP + GFP+). For each colony, 50,000 events were analyzed using a BD Fortessa cytometer.

## Enrichment and sequencing of protein-associated nascent (eSPAN)

The yeast strains WT (cyc1023), *hxk2Δ* (cyc1025), WT + PSF-*HXK2* (cyc1022), and *hxk2Δ* + PSF-*HXK2* (cyc1024) were used in this experiment. Briefly, yeast cells were grown in SC-URA- medium to exponential growth phase (~ 0.5 OD at 600 nm). Two doses of α factor (5 μg/ml; EZBiolab) were added for 3 hours at 25° C to arrest cell growth at the G1 phase. Cells were washed twice with cold water and then released into fresh YPD medium containing 400 mg/L BrdU and 200 mM hydroxyurea for 45 min at 30° C. Hydroxyurea stalls the replication fork but does not interfere with assembly of newly synthesized and parental histones (19, 20).

Cells were fixed by adding freshly prepared paraformaldehyde (final concentration 1%) at 25° C for 20 min, followed by quenching with 0.125 M glycine for 5 min at room temperature. After fixation, cells were washed twice with cold water and collected by centrifugation at 3000 rpm for 5 min. For chromatin immunoprecipitation (ChIP), cells were washed and lysed in 0.1 ml ChIP lysis buffer [50 mM HEPES (pH 8.0), 150 mM NaCl, 2 mM EDTA, 1% Triton X-100, 0.1% sodium deoxycholate] with glass beads. Broken cells were collected and washed twice with NP buffer [1.6 M sorbitol, 2 mM CaCl2, 5mM MgCl, 50 mM NaCl, 14 mM b-mercaptoethanol, 10 mM Tris-HCl (pH 7.4), 0.075% NP-40, 5 mM spermidine]. Chromatin was digested primarily to di- and mononucleosomes using the proper amount of MNase (LS004797, Worthington, ~ 1 unit) at 37° C for 20 min. The digestion was terminated with 5 μl 0.5 M EDTA and 90 μl 5X ChIP lysis buffer and kept on ice for 30 min. Next, cells were lightly sonicated for five cycles (Bioraptor Pico machine, 30 sec ON/OFF) at 4°C to release chromatin fragments into solution. Soluble chromatin was immunoprecipitated with anti-H3K4me3 antibody (ab8580 Abcam) (17). Prewashed Protein G Sepharose beads (17-0618-02, GE Healthcare) were used to recover the immunoprecipitated chromatin. After washing the beads extensively, ChIP DNA was recovered using the Chelex-100 protocol (21).

ChIP DNA was denatured by incubating it at 100° C for 5 min and then on ice for 5 min. DNA was diluted with BrdU IP buffer [1X PBS, 0.0625% Triton X-100 (v/v)]. BrdU antibody (0.17 μg/ml; 555627, BD Biosciences) was added, and samples were incubated at 4° C for 2 hours. Next, 20 μl prewashed Protein G beads (17-0618-02, GE Healthcare) were added to each sample and incubated for an additional hour at 4° C. The beads were extensively washed, then DNA was eluted with 100 ul 1X TE buffer containing 1%

SDS and purified using a QIAGEN MinElute PCR Purification kit. Single-stranded DNA libraries were prepared using an Accel-NGS 1S Plus DNA library kit (10096, Swift Biosciences).

## Sequence mapping and data analysis

Sequence mapping, nucleosome mapping, and eSPAN analysis were performed similarly to what has been previously described (19, 20). Briefly, reads were mapped to the *Saccharomyces* Genome Database (http://www.yeastgenome.org/) reference genome with Bowtie2 software (22). Only paired-end reads with both ends mapped correctly were selected for continued analysis. We determined nucleosome occupancy using 120–170 bp DNA fragments calculated from paired-reads, using Python programs we developed ourselves. To calculate the eSPAN bias pattern, we separated forward (Watson strand) and reverse (Crick strand) reads following the reference genome. Nucleosome positions around DNA replication origins were determined previously (23). Total eSPAN sequence reads at ± 10 nucleosomes surrounding the DNA replication origins were counted. The log2 ratio of Watson over Crick strand reads at each nucleosome position was used to obtain the average eSPAN bias pattern.

## Western blot to detect yeast Rad53-P

The sample collection and process with Rad53-P followed previous publication (40). Blotting was then performed with anti- Rad53 antibody (ab104232, Abcam).

### Results

#### Human HK1 or HK2 cannot complement yeast HXK deletion mutant, as determined by yeast cell growth

In yeast, two *HXK*s, *HXK1* and *HXK2*, and one glucokinase (*GLK1*) phosphorylate glucose, the first irreversible step in the intracellular metabolism of glucose (11). Among these three enzymes, *HXK2* is the dominant player in cell growth, as evidenced by the slow growth phenotype exhibited by *hxk2Δ* and *hxk1Δ*, *hxk2Δ* mutant cells; the growth of *hxk1Δ* cells is comparable to that of wild-type (WT) cells (Fig. 1A, *left*). Importantly, as shown previously (24), the *HXK* inhibitor 2-DG specifically targets *Hxk2* but not *Hxk1* (Fig. 1A, *right*). This conclusion was based on the observation that the *hxk1Δ* cells, grows weaker than in *hxk2Δ* cells on the 2-DG plate (Fig. 1A, *right*).

To investigate the effects of *HXK* expression, we overexpressed both yeast *HXK*s (y*HXK1*, y*HXK2*) and their human orthologs (h*HK1*, h*HK2*) in *hxk1Δ hxk2Δ* double mutant yeast cells. The *HXK* genes were placed under the control of the yeast constitutive *TEF1* promoter (21) in a plasmid, and we grew each transformed strain under both conditions that selected for the plasmid (AA-URA medium) and did not select for the plasmid (YPD medium). We hypothesized that expressing both human and yeast versions of the *HXK* genes could complement the slow growth phenotype of *hxk1Δ hxk2Δ* double mutant. Surprisingly, overexpressing human *HKs* in the yeast mutants cultured in YPD or AA-URA media had no discernible impact on yeast proliferation relative to vector-only control yeast (Fig. 1B). This outcome strongly suggests that human *HK1* and *HK2* do not function effectively in yeast cells. By contrast, expression of yeast *HXK1* and *HXK2* exhibited a complementary effect on yeast proliferation, evident in both YPD and AA-URA media (Fig. 1B). At here, we want to explain a little about the media difference. Because cell could frequently lose the vectors (HXKs-containing or empty control) during proliferation process, the cells on the non-selective media (YPD) are mixture of cells with or without vectors. On the selective media (AA-URA), only vectors containing cells can grow.

Next, we investigated the effects of chemically inhibiting the overexpressed *HXK*s. The purpose is to further test the 2-DG's specificity on the *Hxk*s. When the transformed yeast strains were cultured on YPD supplemented with 0.2% 2-DG (non-selective media with an *Hks* inhibitor), no significant differences in proliferation were observed relative to the vector control strain. This finding can likely be explained by loss of the transformed plasmid under the non-selective conditions. When the strains were cultured on AA-URA + medium supplemented with 0.2% 2-DG (selective media with an *Hks* inhibitor), the inhibitory effect of 2-DG was specific to cells containing y*HXK2* (Fig. 1B). This result underscores the pivotal role played by yeast *HXK2* in cellular proliferation and highlights the specificity of 2-DG targeting y*Hxk2p*.

#### Impact of HXK2 expression on global histone modification levels

To explore the relationship between *HXK2* expression and the chromatin structure/epigenome, we employed four yeast strains characterized by varying *HXK2* expression levels (ranked highest to lowest): WT + *HXK2*, *hxk2Δ* + *HXK2*, WT, and *hxk2Δ* (Fig. 2). First, we confirmed that expression levels for the strains were as expected (Fig. 2A). Overexpression of yeast *HXK2* in the *hxk2Δ* yeast strain significantly upregulated proliferation (Fig. 2A on AA-URA plate), indicating complementation *hxk2Δ*, whereas overexpression of yeast *HXK2* in the WT yeast strain appeared to have no beneficial impact on cell proliferation. The results are consistent with our former observation that the yeast *HXK2* expression can complement the yeast *hxk1Δ hxk2Δ* (Fig. 1).

Next, we conducted immunoblot analysis to assess the effects of *HXK2* expression on global histone modifications, as well as the abundance of the major chromatin silencing protein, Sir2, in the four yeast strains (11). We examined modifications such as H3K36me3, H3K27Ac (associated with active gene transcription), H3K56AC, and H4K5AC (linked to specific histone functions) (25). As the *HXK2* expression level may affect the epigenome, which may show different histone modification levels. However, no substantial differences were detected in the levels of these histone marks or in the abundance of Sir2 across the four yeast strains (Fig. 2B). This finding indicates *HXK2* expression does not exert global effects on histone tail modifications. It is important to note, however, that these results do not rule out the possibility of specific changes in histone modifications occurring at particular loci, warranting further investigation into the potential context-dependent impact of *HXK2* on histone modifications.

#### Impact of HXK2 expression on parental histone transfer

One crucial aspect of stable epigenetic inheritance is the successful replication of epigenetic information during DNA replication. Nucleosome assembly, including the appropriate transfer of both parental and newly synthesized histones, plays a pivotal role in this process. Given the energy-intensive nature of this step, we investigated whether *HXK2* expression influences parental histone transfer and assembly. In WT

yeast, parental H3-H4 tetramers are normally distributed nearly evenly between the leading and lagging strands of DNA replication forks, with a slight bias toward the lagging strand (19). To assess the impact of *HXK2* expression on parental histone distribution, we conducted an eSPAN assay, employing H3K4me3 as a marker for parental H3-H4 histones, as in our previous studies (13, 26). Briefly, yeast cells were synchronized at the G1 phase and released into early S phase in the presence of BrdU and hydroxyurea. Cell harvesting occurred 45 minutes after release (Fig. 3A). Throughout the cell culture process, we employed selective culture medium (AA-URA) to prevent the loss of the *HXK2*-containing plasmid. FACS analysis of DNA content confirmed the effectiveness of our cell synchronization procedure (Fig. 3B), and Western blot analysis confirmed the well-controlled expression of *HXK2* (Fig. 3C). In the hypothesis, we would expect to observe the changes of parental histone distribution pattern on the two replicating strands by eSPAN analysis.

H3K4me3 ChIP, BrdU-IP-ssSeq, and H3K4me3 eSPAN sequence data were mapped to both the Watson and Crick strands of the yeast reference genome. No substantial differences were observed among the four strains for H3K4me3 ChIP and BrdU-IP-ssSeq data at typical mapping sites, such as ARS1309 (Fig. 4A, 4B). H3K4me3 eSPAN peaks at ARS1309 (Fig. 4C) displayed a nearly symmetric bias pattern, with a slight preference for the lagging strand in all four yeast strains. These findings suggest that parental histones H3-H4 were distributed in a nearly symmetrical manner between the leading and lagging strands at hydroxyurea-stalled replication forks, regardless of *HXK2* expression levels.

To determine whether the results we obtained at ARS1309 were characteristic of replication origins around the genome, we calculated the average strand bias ratio of H3K4me3 eSPAN peaks surrounding all 134 early DNA replication origins in yeast. The average bias ratio reflects the relative abundance of histones on the leading vs lagging strand. Consistent with our findings for H3K4me3 eSPAN data at ARS1309 (Fig. 4C), all four strains exhibited a bias toward the lagging strand at these early DNA replication origins, regardless of *HXK2* expression levels (Fig. 4D-G). However, we did observe significant variation in the average strand bias ratio at different nucleosome locations around the replication origins, particularly in the *hxk2Δ* strain (Fig. 4F, G). This variability may be attributable to differences in cell growth rates or low BrdU incorporation rates, factors that can significantly influence eSPAN bias calculations (26). Overall, we conclude *HXK2* expression has only a minor effect on the parental histone transfer process.

#### Effect of HXK2 expression and inhibition on chromatin stability

Even the *HXK2* expression level does not has any clear impact on the parental histone transfer process by, it could affect the chromatin stability by other ways. To test this possibility genetically, we performed a CRASH assay to monitor the transient loss of heterochromatin silencing at the *HML* locus. Flow cytometry analysis was utilized to quantify the rate of silencing loss in each of the four yeast strains (Fig. 2–4). We hypothesized that overexpression or deletion of *HXK2* increases chromatin stability because the glucose metabolism alteration. *HXK2* overexpression (WT + *HXK2*) and deletion (*hxk2Δ* or *hxk2Δ* + *HXK2*) both resulted in a lower silencing loss rate than observed in the WT strain (Fig. 5A, B).

When WT strains were treated with 2-DG to inhibit *HXK2*, the silencing loss rate increased significantly by student t-test (Fig. 5B). In summary, these results indicate that abnormal *HXK2* levels reduce yeast chromatin instability. However, 2-DG treatment can increase chromatin instability. They also shed light on the potential of 2-DG to modulate chromatin dynamics in yeast cells, underscoring the multifaceted effects of this *HXK2* inhibitor on cellular processes.

#### Impact of HXK2 inhibition on cell cycle progression and the DNA damage response

To gain further insights into the effects of *HXK2* inhibition by 2-DG on cell physiology, we assessed its influence on cell cycle progression and its potential to induce systemic DNA damage. First, we investigated the effects of *HXK2* inhibition on cell cycle progression. Logarithmically growing yeast cells (both WT and *hxk2Δ*) were subjected to 2-DG treatment, and we conducted FACS at various time points to measure changes in DNA content. We hypothesized that the cell cycle is predominately arrested at one of cell cycle stages because the glucose metabolism is inhibited. In the WT yeast strain, characterized by normal *HXK2* expression, 2-DG treatment predominantly led to cell cycle arrest at the G2 phase (Fig. 6A). No significant effects were observed in *hxk2Δ* yeast cells following 2-DG treatment; the G1 enrichment observed in this strain at the 8-hour time point was attributed to prolonged culture and nutrient depletion.

Because the 2-DG treatment changed the cell cycle progress, which is frequently associated with DNA damage response, we next investigated whether inhibiting *HXK2* induced systemic DNA damage. Rad53, a vital cell cycle checkpoint protein, is activated and phosphorylated (p-Rad53) in response to DNA replication stress. Thus, p-Rad53 indicates a systemic DNA damage response (27). For instance, exposure to methyl-methanesulfonate induces detectable levels of p-Rad53 (Fig. 6B). We analyzed p-Rad53 levels in strains treated with 2-DG via Western blot analysis. Our results indicate that neither 2-DG-treated WT nor 2-DG-treated *hxk2Δ* yeast strains displayed activated Rad53 (Fig. 6B). These findings suggest that 2-DG-induced cell cycle arrest occurs primarily at the G2 phase but does not lead to widespread DNA damage. These observations provide valuable insights into the specific impact of 2-DG on cell cycle dynamics and establish the absence of a prominent DNA damage response, further contributing to our understanding of the cellular effects of this glycolytic inhibitor.

### Discussion

In this study, we explored the impact of altering expression of the glycolytic enzyme *HXK2* on parental nucleosome transfer and epigenetic stability in yeast. The nucleosome assembly pathway is an energy-intensive process (11). Epigenetic enzymes employ several metabolic intermediates as substrates or co-factors to carry out post-translational modifications of DNA and histones (9), and numerous studies have underscored the crucial role of histone tail acetylation in nucleosome assembly (28, 29). Disrupting acetylation modifications can result in genome instability (28, 29). Thus, we hypothesized that altering *HXK2* expression might impact glycolysis rates in the cell and thus the cell’s global epigenetic signature and genome stability. However, our findings indicate that changes in *HXK* expression, whether resulting from overexpression or deletion, have a minimal effect on parental nucleosome assembly and seem to decrease chromatin instability. Surprisingly, inhibiting *HXK2* via treatment with 2-DG increased chromatin instability.

These findings imply that in yeast cells with *HXK2* deletions, the minimal ATP levels generated through glycolysis, driven by alternative *HXK*s, such as *HXK1* or *GLK1*, are sufficient to sustain epigenetic stability. Interestingly, recent research has shown that a high-glucose culture medium can enhance chromatin instability, with the NAD-dependent histone deacetylase Sir2 playing a role in this process (30). It appears that energy availability, rather than enzyme levels, is the primary rate-limiting factor for chromatin stability.

The proliferation of many cancer cells heavily relies on aberrant energy metabolism, such as the Warburg effect or aerobic glycolysis (31). Therefore, inhibiting Warburg effect has emerged as a potential strategy to target and eliminate tumor cells, with the *HXK* inhibitor 2-DG being one potential candidate drug of this type. As mentioned, our study suggests that 2-DG treatment increases epigenetic instability. However, this effect appears to occur independent of glycolysis inhibition, as lower *HXK2* levels increase epigenetic stability. Thus, the precise mechanism underlying 2-DG's impact on chromatin stability warrants further investigation.

In our experiments, overexpressing the human-derived *hHK1* and *hHK2* genes did not rescue the growth defect phenotype of a yeast *hxk1Δ hxk2Δ* mutant, despite yeast *HXK1* and *HXK2* genes in the same vector functioning effectively. This observation suggests the slow growth phenotype of the *hxk2Δ* strain may not solely result from its reduced glucokinase activity. Alternatively, h*HK1* and h*HK2* may require additional factors to function that are absent in yeast cells. Interestingly, the human GlkB (h*HK*4) protein, which has a similar molecular weight as the yeast *HXK2* protein, can complement the growth phenotype of a yeast *hxk1Δ*/*hxk2Δ*/glk1Δ mutant (32). Likewise, several plant *HXK*s have been shown to complement growth phenotypes in yeast *HXK* mutants (33–35).

## Conclusions

Hexokinases (*HXK*s) are the key enzymes regulating glycolysis in cells. Overexpression of *HXK*s has been linked to numerous types of cancers and targeting *HXK*s has been suggested as a potential strategy of cancer therapy. In this study, our result suggests the altered *HXK*s expression level decreases the chromatin instability. However, 2-Deoxy-D-glucose (2-DG), a *HXK* inhibitor, treatment cells show increased chromatin instability with unknown mechanisms.

## Declarations

**Ethics approval and consent to participate.** Not applicable.

**Consent for publication.** Not applicable.

**Availability of data and materials.** The datasets generated and analyzed during the current study are available in the GEO repository, GSE 245005.

**Competing interests.** The authors declare that they have no conflict of interest.

**Authors' contributions.** SK, CY and DY conceived the project and designed the study. SK, CY and JJ performed experiments and data analysis. QD and HG did bioinformatic data analysis. SK, CY, ZZ and HG analyzed the data and interpreted the outcomes. All authors participated in reviewing and interpretation of the data and writing the manuscript. All authors read and approved the final manuscript.

**Acknowledgements.** We thank Dr. Yi Yang, Jizhi Ge, and Huipeng Liu for laboratory assistance and Dr. Xia Zhao for biostatistics analysis. We also thank Kristin Harper, PhD, of Harper Health & Science Communications, LLC, for providing editorial support in accordance with Good Publication Practice guidelines.

**Funding.** This work was supported by NIH grant R01GM130588 (to C.Y.), the Hormel Startup Fund, and the National Natural Science Foundation of China (Grants No. 32090031, 32070610, to H.G.).

## Figures

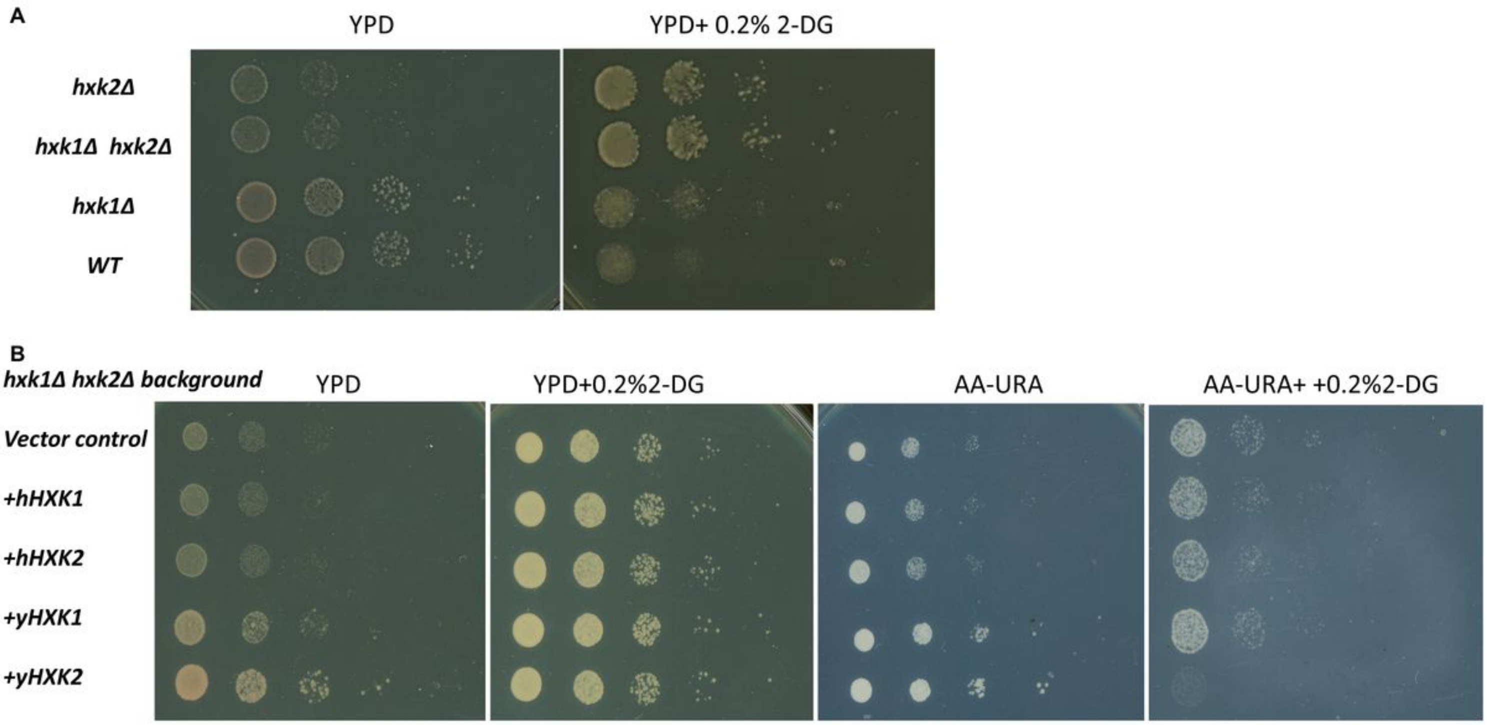

### Figure 1

**In yeast, *HXK2* is the dominant hexokinase; overexpressing yeast *HXK1* or *HXK2*, but not human *HXK1* or *HXK2*, complements the slow cell growth phenotype in yeast cells lacking *HXK1*or *HXK2*.** (A) Ten-fold serial dilutions of yeast cells of the indicated genotypes were plated onto YPD medium with or without 0.2% 2-deoxy-D-glucose (2-DG, an *HXK2* inhibitor). This result suggests that *HXK2* is the dominant player in glucose metabolism. (B) Yeast *HXK1* or *HXK2* can complement the cell growth phenotype in *hxk1Δ hxk2Δ*cells, but human *HXK1* and *HXK2* cannot. Ten-fold serial dilutions of yeast cells of the indicated genotypes were plated onto YPD medium with or without 0.2% 2-DG (nonselective for the plasmid containing the *HXK* genes) and AA-URA medium with or without 0.2% 2-DG (selective for the plasmid). These results suggest that the 2-DG is specifically targeting *HXK2* and that overexpression of *HXK1* can partially complement the growth phenotype of *HXK2* deletion, but 2-DG has no effect on the *HXK1*.

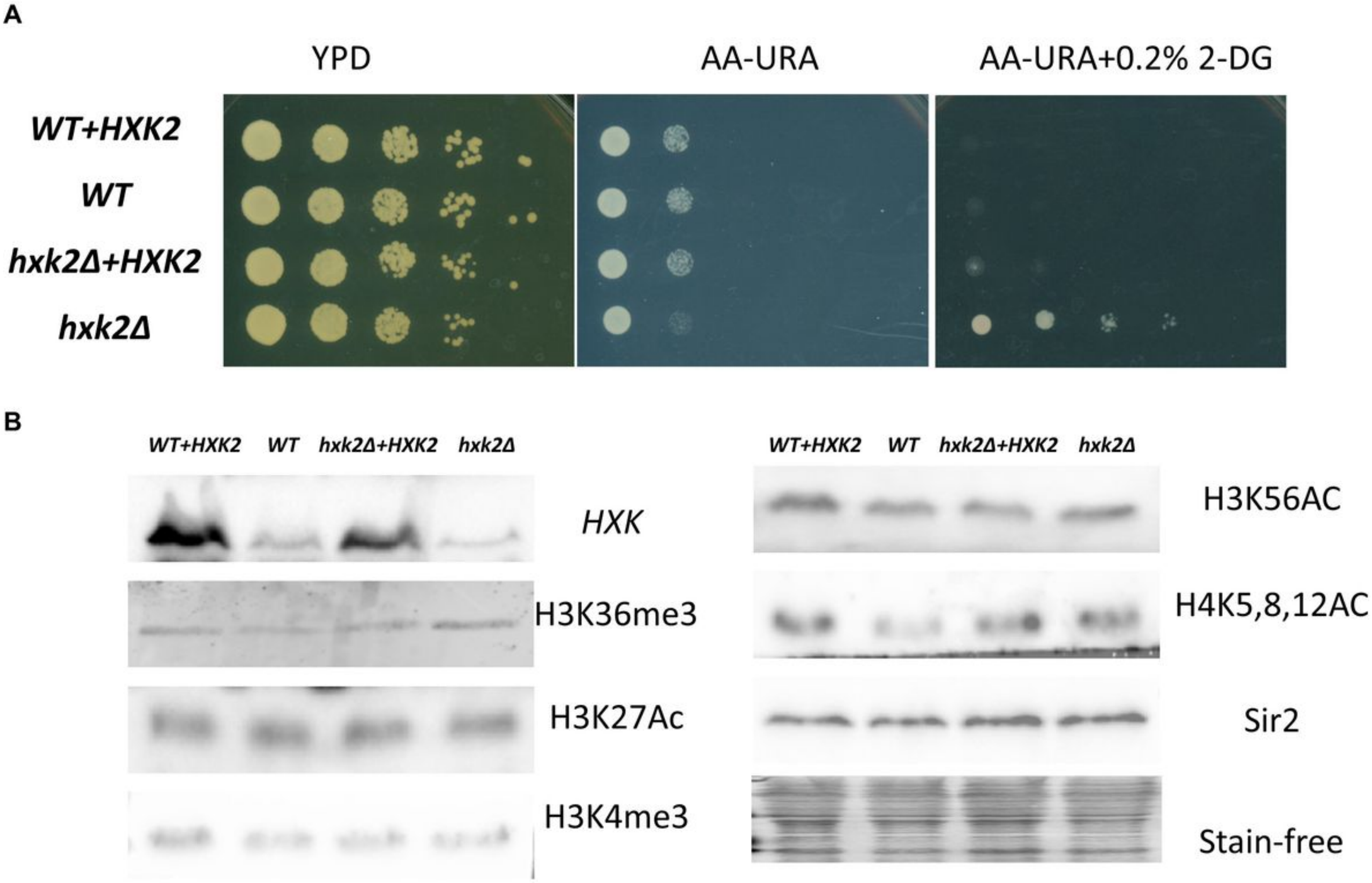

### Figure 2

**Impact of *HXK2* expression on yeast cell growth and total histone modification levels.** In this panel, we confirm the *HXK*s expression level by western blot and test *HXK*s level on total histone modification levels. (A) Cell growth rate of WT, WT+*HXK2*, *hxk2Δ*+*HXK2*, and *hxk2Δ* yeast strains. Ten-fold serial dilutions of yeast cells were plated onto YPD medium and AA-URA (conditions selective for the vector containing cells) with or without 0.2% 2-deoxy-D-glucose (2-DG, an *HKs*inhibitor) medium. The purpose of these dot assay is phenotype confirming of the genotypes. (B)Total levels of histone modifications (H3K36me3, H3K27Ac, H3K4me3, H3K56Ac and H4K5,8,12Ac) has subtle change in the yeast strains from (A) with Western blot analysis. The exposure growth cells on AA-URA medium were collected for Western blot analysis.

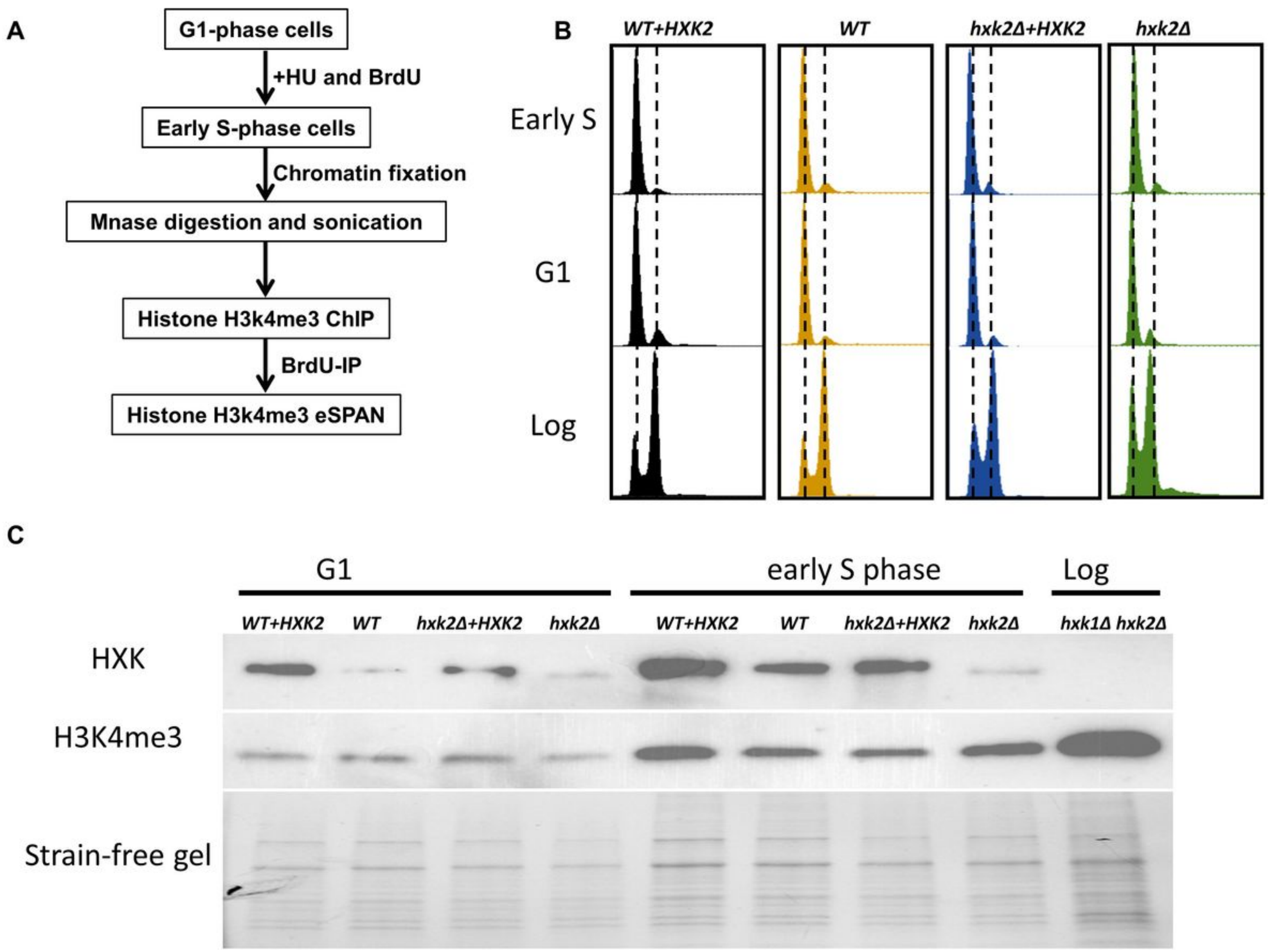

### Figure 3

**Parental histone transfer experimental procedure: H3K4me3 eSPAN (enrichment and sequencing of protein-associated nascent DNA) analysis.** (A) Procedure for monitoring the deposition of parental (H3K4Me3) histones at early DNA replication origins in yeast. (B) Fluorescence-activated cell sorting (FACS) analysis monitoring cell cycle progression in WT, WT+*HXK2*, *hxk2Δ*+*HXK2*, and *hxk2Δ* yeast strains. The FACS data indicate that the cell synchronization procedure for eSPAN analysis work well. (C) Western blot analysis monitoring *HXK*and H3K4me3 levels in the yeast strains from (B). The log phase *hxk1Δ hxk2Δ* strain sample serves as a negative control for the *Hxk* antibody. This data suggests the *HXK2* expression level is well controlled.

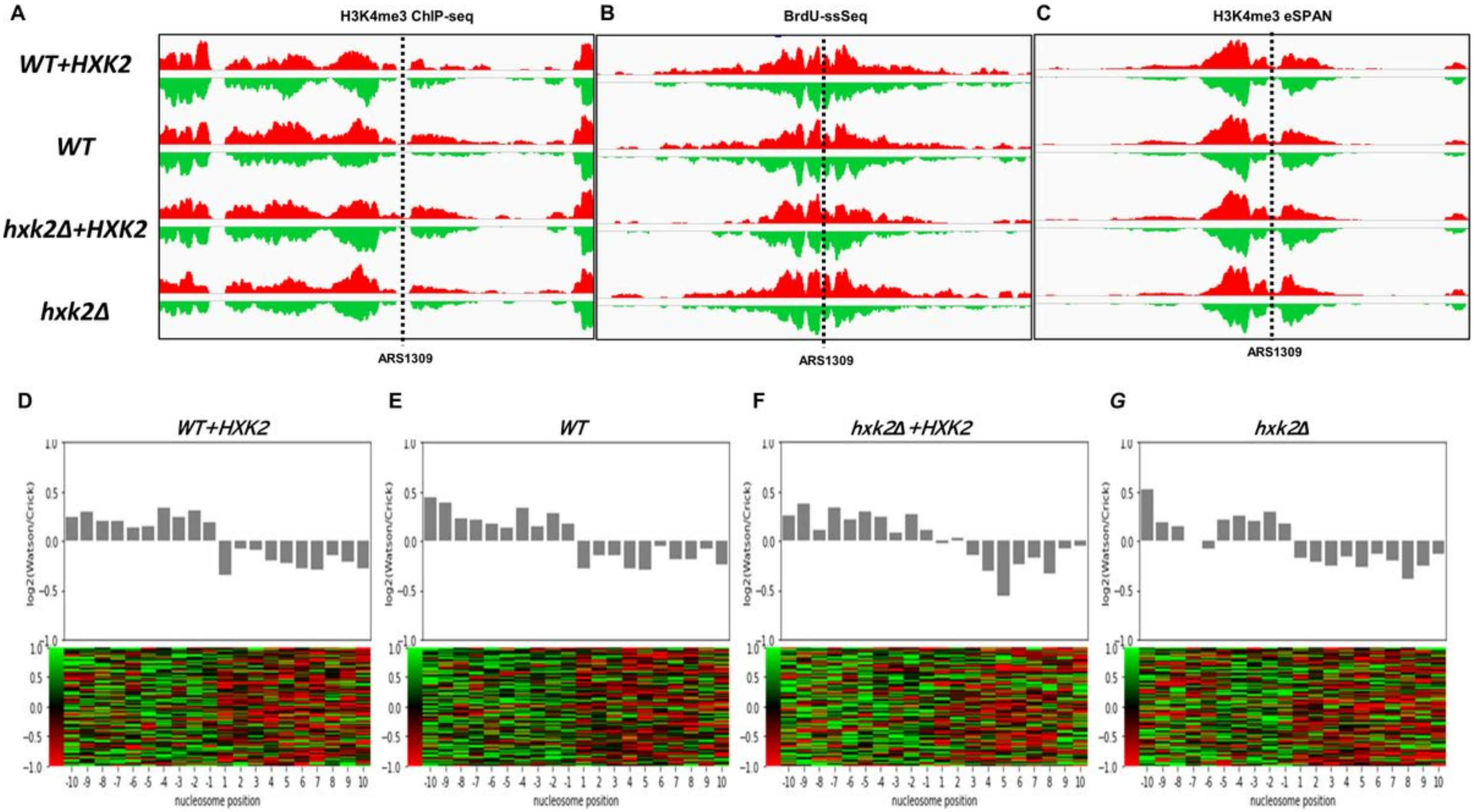

### Figure 4

**Parental histone H3 (H3K4Me3) eSPAN (enrichment and sequencing of protein-associated nascent DNA) analysis at different *HXK2* expression levels.** A snapshot of parental histone (A) H3 (H3K4me3) chromatin immunoprecipitation (ChIP), (B) BrdU-IP-ssSeq, and (C) H3K4me3 eSPAN read enrichment at the early replication origin ARS1309 for the WT, WT+*HXK2*, *hxk2Δ*+*HXK2*, and *hxk2Δ* yeast strains. Sequence reads were mapped to both the Watson (*red*) and Crick (*green*) strands of the reference genome. We did not observe any obvious difference in the BrdU IP-ssSeq, H3K4me3 ChIP-seq and eSPAN. (D-G) Average bias ratio of parental histone H3 (H3K4Me3) for the WT, WT+*HXK2*, *hxk2Δ*+*HXK2*, and *hxk2Δ* yeast strains. *Top*: Average bias ratio of H3K4Me3 eSPAN peaks at each of the 10 nucleosomes surrounding the 134 early replication origins in the yeast genome. *Bottom*: Heatmaps representing the bias ratio of the same H3K4Me3 eSPAN peaks. Individual nucleosome positions (−10 to +10) are indicated relative to the origin. Each row represents the average log2 Watson/Crick ratio of H3K4Me3 eSPAN sequence reads at one origin. Although there are some variations in the bias, we did not observe any consistent obvious difference between different strains.

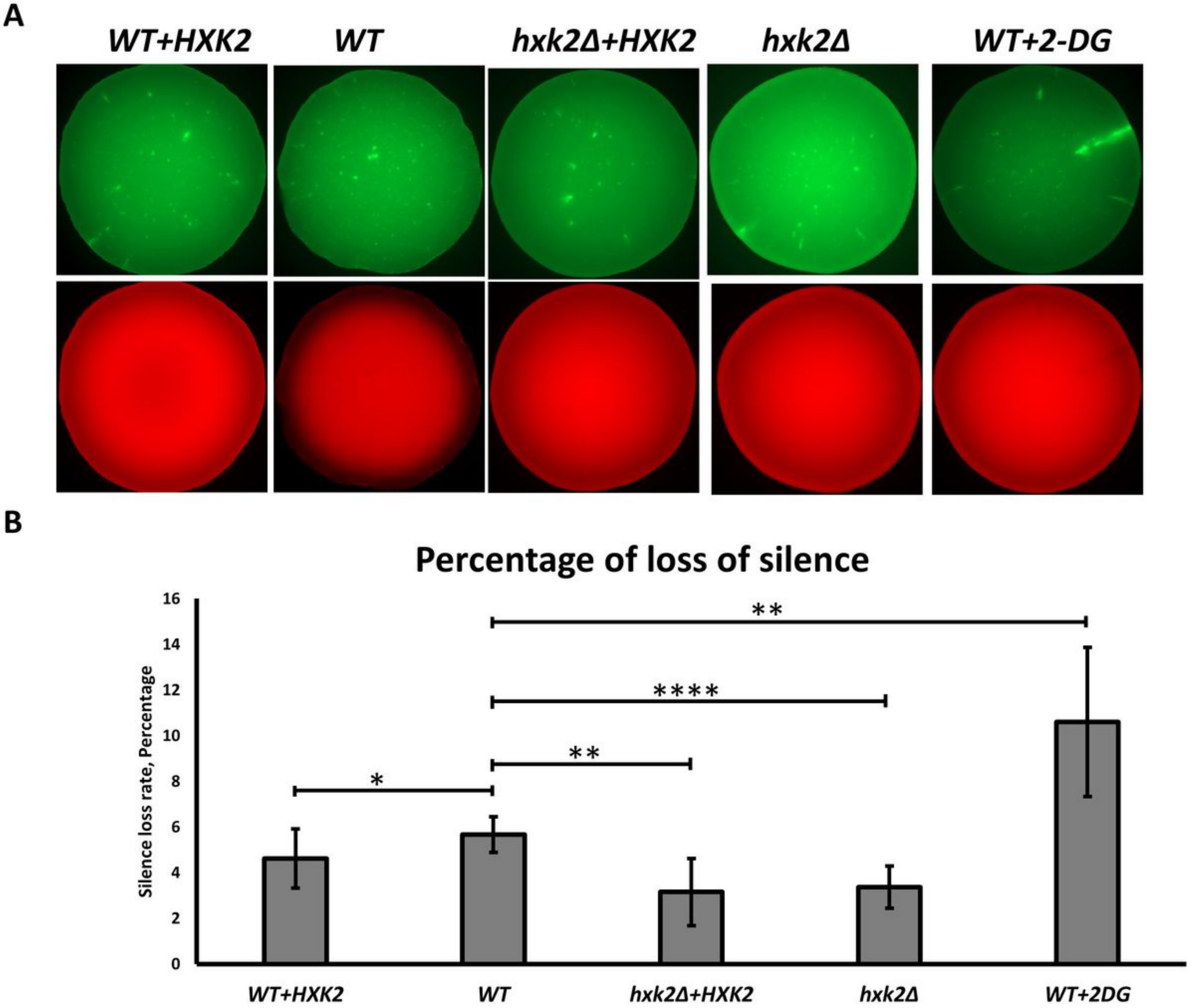

### Figure 5

**Effect of Hxk levels on loss of silencing at the *HML* locus.** (A) Typical fluorescence images of colonies derived from WT, WT+*HXK2*, *hxk2Δ*+*HXK2*, *hxk2Δ* yeast strains containing the LoxP-RFP-LoxP-GFP cassette at URA3 locus and a HMLα::cre at the silenced HML locus. The CRASH (Cre-reported altered states of heterochromatin) system (36) was used in this assay. Bright green sectors in GFP channels or dark sectors in RFP channels represent loss of silencing. (B) The WT, WT+*HXK2*, *hxk2Δ*+*HXK2*, and *hxk2Δ* yeast strains showed different degrees of silencing at the *HML* locus when analyzed by the CRASH (Cre-reported altered states of heterochromatin) assay. Error bar = standard error. *p<0.05, **p<0.01, and ****p < 0.0001 by Student's t-test. The result indicates that 2-DG treatment increases the chromatin instability.

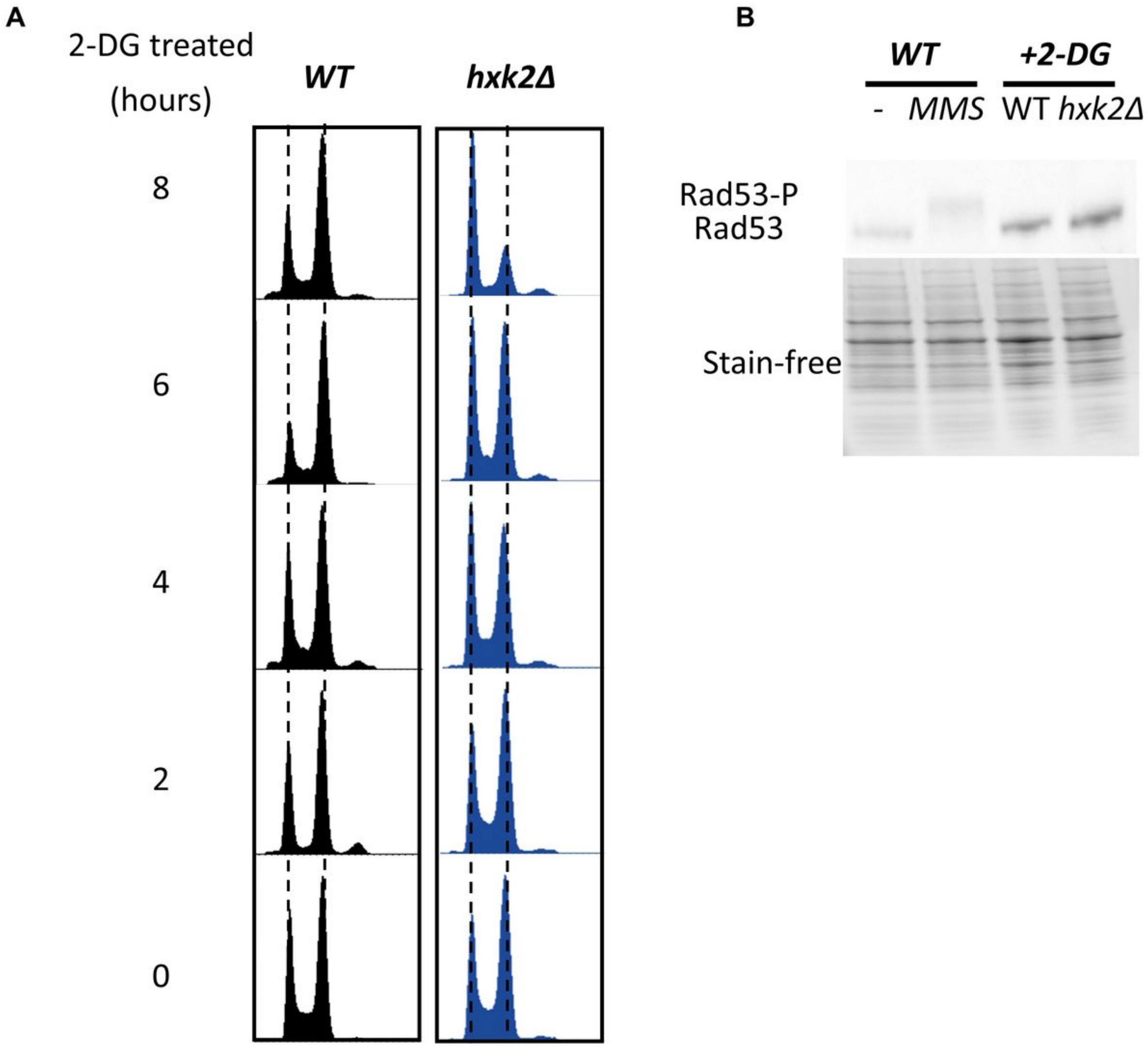

**Figure 6**

**Hxk inhibition arrests yeast cells at G2 phase, but does not activate the cell cycle checkpoint.** (A) Fluorescence-activated cell sorting (FACS) analysis of DNA content in WT and *hxk2Δ* yeast strains treated with 0.2% 2-deoxy-D-glucose (2-DG, an Hxk2 inhibitor) at different time points. This data indicates that 2-DG treatment leads to more G2 cells. (B) Immunoblot analysis of levels of Rad53-P, a cell cycle checkpoint protein and marker of the DNA damage response, in WT and *hxk2Δ* yeast strains. WT yeast cells before and after treatment with methylmethane sulphonate (MMS; a DNA damaging agent) serve as negative and positive controls, respectively. This data indicates that the 2-DG treatment do not lead to systemic DNA damage response. For 2-DG-treated samples, WT and *hxk2Δ* cells were treated with 0.2% 2-DG for 2 hr.

## Supplementary Files

This is a list of supplementary files associated with this preprint. Click to download.

- Suppledata.docx
